# Remodeling of the brain correlates with gait instability in cervical spondylotic myelopathy

**DOI:** 10.3389/fnins.2023.1087945

**Published:** 2023-02-02

**Authors:** Xianyong Wu, Ying Wang, Jianchao Chang, Kun Zhu, Siya Zhang, Yan Li, Junxun Zuo, Senlin Chen, Weiming Jin, Tingfei Yan, Kun Yang, Peng Xu, Peiwen Song, Yuanyuan Wu, Yinfeng Qian, Cailiang Shen, Yongqiang Yu, Fulong Dong

**Affiliations:** ^1^Department of Orthopedics, The First Affiliated Hospital of Anhui Medical University, Hefei, China; ^2^Department of Spine Surgery, The First Affiliated Hospital of Anhui Medical University, Hefei, China; ^3^Department of Radiology, The First Affiliated Hospital of Anhui Medical University, Hefei, China; ^4^School of Basic Medical Sciences, Anhui Medical University, Hefei, China; ^5^Department of Orthopedics, Dongcheng Branch of The First Affiliated Hospital of Anhui Medical University (Feidong People’s Hospital), Hefei, China; ^6^Department of Medical Imaging, The First Affiliated Hospital of Anhui Medical University, Hefei, China

**Keywords:** resting-state fMRI, diffusion tensor imaging, cervical spondylotic myelopathy, gait instability, caudate nucleus, corticospinal tract

## Abstract

**Introduction:**

Cervical spondylotic myelopathy (CSM) is a common form of non-traumatic spinal cord injury (SCI) and usually leads to remodeling of the brain and spinal cord. In CSM with gait instability, the remodeling of the brain and cervical spinal cord is unclear. We attempted to explore the remodeling of these patients’ brains and spinal cords, as well as the relationship between the remodeling of the brain and spinal cord and gait instability.

**Methods:**

According to the CSM patients’ gait, we divided patients into two groups: normal gait patients (nPT) and abnormal gait patients (aPT). Voxel-wise z-score transformation amplitude of low-frequency fluctuations (zALFF) and resting-state functional connectivity (rs-FC) were performed for estimating brain changes. Cross-sectional area (CSA) and fractional anisotropy (FA) of the spinal cord were computed by Spinal cord toolbox. Correlations of these measures and the modified Japanese Orthopedic Association (mJOA) score were analyzed.

**Results:**

We found that the zALFF of caudate nucleus in aPT was higher than that in healthy controls (HC) and lower than that in nPT. The zALFF of the right postcentral gyrus and paracentral lobule in HC was higher than those of aPT and nPT. Compared with the nPT, the aPT showed increased functional connectivity between the caudate nucleus and left angular gyrus, bilateral precuneus and bilateral posterior cingulate cortex (PCC), which constitute a vital section of the default mode network (DMN). No significantly different FA values or CSA of spinal tracts at the C2 level were observed between the HC, nPT and aPT groups. In CSM, the right paracentral lobule’s zALFF was negatively correlated with the FA value of fasciculus gracilis (FCG), and the right caudate zALFF was positively correlated with the FA value of the fasciculus cuneatus (FCC). The results showed that the functional connectivity between the right caudate nucleus and DMN was negatively correlated with the CSA of the lateral corticospinal tract (CST).

**Discussion:**

The activation of the caudate nucleus and the strengthening functional connectivity between the caudate nucleus and DMN were associated with gait instability in CSM patients. Correlations between spinal cord and brain function might be related to the clinical symptoms in CSM.

## 1. Introduction

Cervical spondylotic myelopathy (CSM) is a common form of non-traumatic spinal cord injury (SCI) in middle- and old-aged populations (Bakhsheshian et al., 2017). The symptoms of CSM include gait instability, hand clumsiness and limb sensory losses. Moreover, gait instability is one of the most common and important symptoms in CSM patients, which can lead to a deterioration of health and living quality because of the high incidence of falls and fractures (Radcliff et al., 2016). Previous studies had revealed that the extremity kinematics and electromyographic characterization of patients with unstable gait were changed (Haddas et al., 2018, 2019).

The chronic compression of the spinal cord causes ischemia and Wallerian degeneration (David et al., 2019; Azzarito et al., 2020; Tu et al., 2021). Degeneration in the spinal cord was also associated with brain function remodeling (Bernabeu-Sanz et al., 2020). These changes in microstructure and function might be aimed at maintaining neurological function (Holly et al., 2007), and the remodeling of the sensorimotor cortex and spinal cord tracts was related to sensorimotor dysfunction in CSM (Chen et al., 2022). In addition, the brain function (e.g., measured by the amplitude of low-frequency fluctuations; ALFF) remodeling contributes to disease prognosis and the partial recovery of the primary motor cortex after decompression surgery proved the existence of plasticity in the brain (Ryan et al., 2018; Zhao et al., 2022b). Nevertheless, in CSM patients, the changes of brain and the spinal cord tracts and their relationship with gait instability remain unclear.

In the present study, we adopted resting-state functional magnetic resonance imaging (fMRI) and DTI to explore the function of the brain and microstructures of spinal tracts and attempted to observe the correlation between gait instability and imaging features in CSM patients. Building on preceding fMRI studies in CSM, we hypothesized that the remodeling of particular brain regions and spinal tracts contributes to gait instability in CSM.

## 2. Materials and methods

### 2.1. Participants

Our study included 32 right-handed CSM patients. According to their gait, we divided the CSM patients into two groups: sixteen normal gait patients (nPT) and sixteen abnormal gait patients (aPT). The following inclusion criteria were used: (1) MRI demonstrated cervical spinal cord compression with a diagnosis of CSM; (2) no history of cervical and craniocerebral trauma or surgery; (3) no history of psychiatric disorders; (4) no peripheral neuropathy; and (5) no contraindications for MRI examination. Sixteen right-handed healthy controls (HCs) were also recruited.

### 2.2. Clinical scale scores

Neurological function was evaluated by two experienced clinicians by the modified Japanese Orthopedic Association (mJOA) scale, choosing the lower extremity subscore of the mJOA describing gait impairment (Kopjar et al., 2011).

### 2.3. MRI data acquisition

#### 2.3.1. Brain image acquisition

A 3.0T MRI (General Electric 750 w, USA) scan with a 24-channel head coil. Earplugs were used to reduce scanner noise, and foam padding was used to minimize head motion. The settings of resting-state blood-oxygen-level-dependent (BOLD) fMRI data were obtained by employing a gradient-echo single-shot echo planar imaging (GRE-SS-EPI) sequence with the following parameters: repetition time (TR) = 2,000 ms; echo time (TE) = 30 ms; FOV = 220 mm × 220 mm; matrix size = 64 × 64; flip angle = 90°; slice thickness = 3 mm, slice gap = 1 mm; 35 interleaved axial slices; 185 volumes. High resolution 3D T1-weighted structural images were required using a brain volume (BRAVO) sequence with the following parameters: TR = 8.5 ms; TE = 3.2 ms; inversion time (TI) = 450 ms; FOV = 256 × 256 mm; matrix size = 256 × 256; flip angle = 12°; slice thickness = 1 mm, no gap; 188 sagittal slices.

#### 2.3.2. Cervical image acquisition

A 1.5T magnetic resonance imaging (MRI) system (Philips Ingenia 1.5T, Holland) was used. Diffusion-weighted imaging was acquired by employing a single-shot spin echo echo-planar image (SS-SE-EPI) sequence with the following parameters: TR = 3,000 ms, TE = 83 ms, FOV = 300 × 300 mm, acquisition matrix = 100 × 98 mm, slice thickness = 3 mm, no gap; 50 slices; 15 gradient directions. The diffusion-weighted coefficients were b = 0 and 800 s/mm^2^, and 16 images were acquired after each scan. Three-dimensional (3D) T2-weighted images were obtained with the following parameters: TR = 2,300 ms, TE = 83 ms, slice thickness = 4 mm, FOV = 150 × 150 mm, acquisition matrix = 176 × 139 mm, number of slices = 60, no gap.

### 2.4. Image processing

Brain fMRI preprocessing was performed by Statistical Parametric Mapping (SPM) 12^1^ and executed in the MATLAB 2013b platform (Mathworks, Sherborn, MA, USA). The procedures included (1) removal of the first 10 time points; (2) slice timing and head motion correction (translational or rotational motion parameters more than 3 mm or 3° were excluded); (3) coregistration of functional data to the structural T1-weighted image and normalization into the Montreal Neurological Institute (MNI) space with a resampling voxel size of 3 × 3 × 3 mm; (4) smooth 6 mm full-width half-maximum Gaussian Kernel;(5) removal of nuisance covariate regression (cerebrospinal fluid signals, white matter signals, and Friston-24 head motion parameters) and linear trend.

ALFF calculation: We used the fast Fourier transform to obtain the power spectrum, calculated the square root at each frequency of the power spectrum and obtained the averaged square root across the 0.01–0.08 Hz frequency range. This averaged square root value was the ALFF value, and zALFF was transformed by z-score transformation and used for subsequent group-level analysis.

Seed-based functional connectivity (FC) calculation: We adopted the seed-to-voxel correlation to calculate the FC value between seeds and the whole brain. We obtained the FC map by analyzing linear correlation between seeds and the other voxels in the whole brain, and zFC map was transformed by Fisher’s z transformation and used for subsequent group-level analysis. Seeds were selected by the zALFF clusters showing a between group differences approach, which was calculated by the “REST Image calculator” in RESTplus V1.27 [Resting State fMRI Data Analysis Toolkit plus (Jia et al., 2019)].

Cervical image preprocessing was performed by the Spinal Cord Toolbox (version 5.3.0) and the PAM50 spinal cord template (De Leener et al., 2017). The procedures included (1) spinal cord segmentation by a deep-learning-based algorithm (Gros et al., 2019); (2) vertebral labeling by manual identification of the C2/3 disk; (3) registration to the PAM50 template by linear and non-linear algorithms; (4) warp the template to match the subject imaging; and (5) computing the cross-sectional area (CSA) and DTI parameters at the C2 level. In addition, we conducted motion correction before processing the DTI data (Xu et al., 2013).

### 2.5. Statistical analyses

#### 2.5.1. Brain function analyses

One way analysis of variance (ANOVA) was performed in HC, nPT and aPT within the gray matter masks, and age and gender were used as covariates, *p* ≤ 0.001 (significance threshold) was corrected for multiple comparisons with familywise error (FWE) correction at the cluster level *via* SPM12. The zALFF values of significantly different brain regions were extracted and performed by one way ANOVA with Bonferroni *post-hoc* test, and these brain regions were chosen as seed regions for FC analyses. None of the participants exhibited abnormalities in brain structures.

#### 2.5.2. Cervical image analyses

Previous studies have shown that several spinal cord tracts are related to locomotion and gait, including the fasciculus gracilis (FCG), fasciculus cuneatus (FCC), lateral corticospinal tract (CST), and spinocerebellar tract (SCT) (Yasuda et al., 1993; Fink, 2013; Chalif et al., 2022; Smith et al., 2022). Considering that the compression of the spinal cord may reduce the measurement accuracy (Hopkins et al., 2018),we chose the spinal cord at the C2 level. Therefore, we computed the CSA and fractional anisotropy (FA) values of those paired tracts at the C2 level by the Spinal Cord Toolbox. One way ANOVA with Bonferroni *post-hoc* test was used to analyze the difference in CSA and FA value between the HC, nPT and aPT groups. Due to the absence of some CSM patients, we finally adopted 6 HC’ and 18 patients’ (10 nPT and 8 aPT) cervical images for analyses.

All statistical analyses were performed using GraphPad Prism 9.0 (San Diego, CA, USA) software^2^. Normality was tested by D’Agostino-Pearson’s. Chi-square test was used for gender difference analysis. One way ANOVA with Bonferroni *post-hoc* test was performed for finding the different CSA, FA value between the HC and nPT/aPT groups. Two-sample *t*-test was used to analyze mJOA score between the nPT and aPT groups. Pearson correlation analysis was used to analyze the correlation between the mJOA scores and zALFF values, as well as their correlation with FC and FA values. When the data did not follow a normal distribution, Mann-Whitney test and the Spearman correlation were used. *P < 0.05; **P < 0.01; ***P < 0.001.

## 3. Results

### 3.1. Demographic data and clinical scale scores

No significant difference was noted in age or gender between the nPT, aPT, and HC groups (*P* > 0.05). No significant difference was found in the symptom duration between aPT and nPT (*P* > 0.05). The mJOA score of nPT was higher than the score of aPT (*P* < 0.001). To evaluate gait impairment, we analyzed the lower extremity subscore of the mJOA. Compared with aPT, nPT showed a higher score in the lower extremity (*P* < 0.001; Mann-Whitney test), and there was no significant difference in the upper extremity, sensation or sphincter score of mJOA between nPT and aPT (*P* > 0.05) (Table 1).

**TABLE 1:** Demographic and clinical characteristics.

	nPT (*n* = 16) mean ± SD	aPT (*n* = 16) mean ± SD	HC (*n* = 16) mean ± SD	*P-*value
Age (years)	51.38 ± 6.91	53.56 ± 9.51	52.63 ± 10.76	0.797
Gender (M/F)	8/8	8/8	8/8	>0.999
CSM duration (month)	15.06 ± 2.87	17.25 ± 3.25	–	0.618
mJOA score	15.31 ± 0.79	12.81 ± 1.23	–	<0.001
mJOA UE subscore	3.56 ± 0.51	3.62 ± 0.62	–	>0.999
mJOA LE subscore	7 ± 0	4.5 ± 0.82	–	<0.001
mJOA sensory subscore	1.75 ± 0.58	1.69 ± 0.48	–	0.741
mJOA sphincter subscore	3 ± 0	3 ± 0	–	>0.999

nPT, normal gait patients; aPT, abnormal gait patients; HC, healthy control; CSM, cervical spondylotic myelopathy; UE, upper extremity; LE, lower extremity. One way ANOVA test was performed among nPT, aPT, and HC groups. Chi-square test was performed for gender difference. Two-sample t-test was performed between nPT and aPT groups. Mann-Whitney test was performed when the data did not follow a normal distribution.

### 3.2. zALFF differences between HC, nPT, and aPT

**FIGURE 1:**
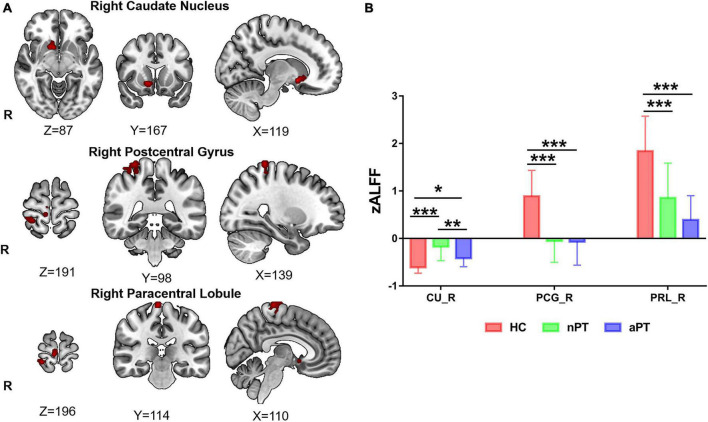
z-score transformation amplitude of low-frequency fluctuations (zALFF) differences between HC, nPT, and aPT groups. **(A)** The right caudate nucleus, right postcentral gyrus, and right paracentral lobule were the significantly different brain regions among the three groups. **(B)** The zALFF values of these significantly different brain regions were extracted and analyzed by one-way analysis of variance (ANOVA) with Bonferroni *post-hoc* test. The caudate zALFF in aPT was higher than that in HC, and lower than that in nPT. The zALFF of the right postcentral gyrus and paracentral lobule in HC was higher than those of aPT and nPT. CU, caudate nucleus; PCG, postcentral gyrus, PRL, paracentral lobule; HC, healthy control; nPT, normal gait patients; aPT, abnormal gait patients. *P < 0.05; **P < 0.01; ***P < 0.001.

The zALFF of the right caudate nucleus, right postcentral gyrus, and right paracentral lobule were significantly different among the three groups (FWE correction, corrected *p* ≤ 0.05 at cluster level; Figure 1A and Table 2). Those significantly changed brain regions were extracted as seeds for subsequent analysis. To explore the differences further, we extracted the zALFF values of the seeds in HC, nPT/aPT and performed one-way ANOVA with Bonferroni *post-hoc* test. The right caudate zALFF value in aPT was higher than that in HC (*p* < 0.05) and lower than that in nPT (*p* < 0.01). The zALFF values of right postcentral gyrus and paracentral lobule were higher than those of nPT and aPT (*p* < 0.001) (Figure 1B). The right caudate nucleus was the only significantly different region between nPT and aPT, which indicated that it might be related to gait instability in CSM.

**TABLE 2:** Regions of significant z-score transformation amplitude of low-frequency fluctuations (zALFF) difference in three groups.

Brain region	MNI coordinates	Cluster voxels	F value
Right postcentral gyrus	27, –39, 72	53	16.4
Right caudate nucleus	12, 9, –9	25	22.05
Right paracentral lobule	6, –27, 75	33	18.81

HC, healthy control; nPT, normal gait patients; aPT, abnormal gait patients; MNI, Montreal Neurological Institute.

### 3.3. Seed-based functional connectivity differences in CSM patients with different gaits

**FIGURE 2:**
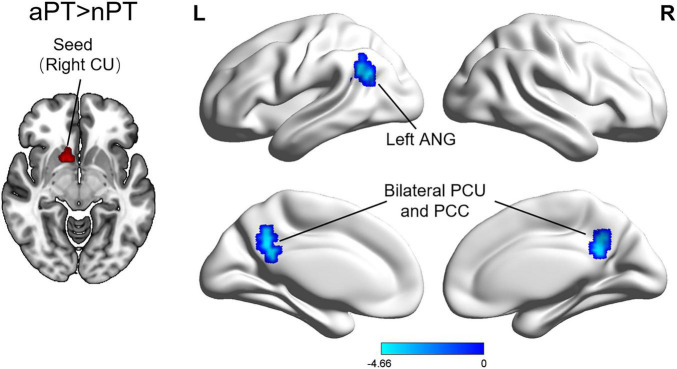
Differences of functional connectivity between the nPT and aPT. Compared with nPT, aPT exhibited increased caudate nucleus connectivity to several brain regions: the left angular gyrus, bilateral precuneus, and bilateral posterior cingulate cortex. nPT, normal gait patients; aPT, abnormal gait patients; CU, caudate nucleus; ANG, angular gyrus; PCU, precuneus; PCC, posterior cingulate cortex.

Compared with the nPT, the aPT showed increased FC between the caudate nucleus and the left angular gyrus, bilateral precuneus, and bilateral posterior cingulate cortex (PCC) (FWE correction, corrected *p* ≤ 0.05 at cluster level) (Figure 2 and Table 3). Interestingly, these brain regions are a part of the default mode network (DMN). No significant group differences were observed in FC with other seed regions.

**TABLE 3:** Regions of significant functional connectivity (FC) difference between abnormal gait patients (aPT) and normal gait patients (nPT).

Seed	Brain region	MNI coordinates	Cluster voxels	T value
	aPT>nPT	–	–	–
Right caudate nucleus	Left angular gyrus	–54, –57, 27	50	–4.51
	Bilateral precuneus/ bilateral PCC	–6, –48, 36	104	–4.66

nPT, normal gait patients; aPT, abnormal gait patients; MNI, Montreal Neurological Institute; PCC, posterior cingulate cortex.

### 3.4. Spinal tract differences in HC and CSM patients with different gaits

**FIGURE 3:**
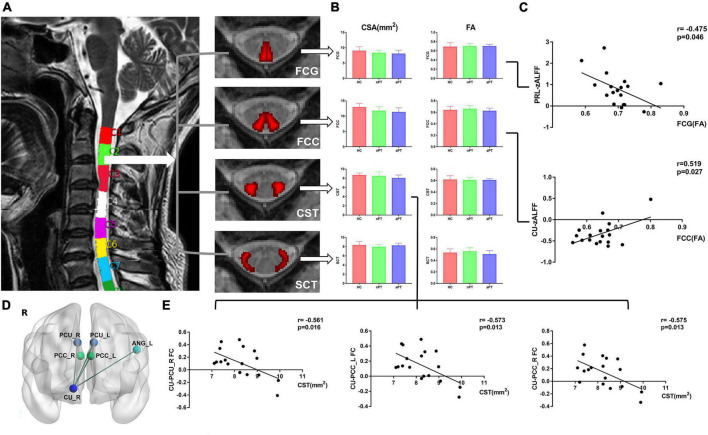
Comparison of spinal tracts and the relationship between fractional anisotropy (FA), cross-sectional area (CSA), z-score transformation amplitude of low-frequency fluctuations (zALFF) value and caudate connectivity. **(A)** The CSA and FA values of paired FCG, FCC, CST, and SCT at the C2 level were computed by the Spinal Cord Toolbox. **(B)** One-way analysis of variance with Bonferroni *post-hoc* test was preformed and no significant difference in the FA value or CSA of these tracts at the C2 level was noted between the HC, nPT, and aPT groups. **(C)** The zALFF of PRL was negatively correlated with the FA value of FCG and the zALFF of CU was positively correlated with the FA value of FCC. **(D)** FC between the right CU and left ANG, bilateral PCU, and bilateral PCC was extracted. **(E)** The CSA of the CST at C2 level was negatively correlated with the FC between the right CU and right PCU and bilateral PCC. FCG, fasciculus gracilis; FCC, fasciculus cuneatus, CST, lateral corticospinal tract; SCT, spinocerebellar tract; FC, functional connectivity; PRL, paracentral lobule; CU, caudate nucleus; ANG, angular gyrus; PCU, precuneus; PCC, posterior cingulate cortex.

We computed the FA value and CSA of the FCG, FCC, CST, and SCT and preformed one-way ANOVA with Bonferroni *post-hoc* test (Figure 3A). No significant difference in the FA value or CSA of these tracts at C2 level was observed between the HC, nPT, and aPT groups (Figure 3B).

To explore the relationship between the spinal cord and brain in CSM, we analyzed the correlation between zALFF and FC values of changed brain regions and CSA and FA values of spinal tracts. In CSM patients, we found that the zALFF value of right paracentral lobule correlated with the streamlines at the FA value of FCG at the C2 level (r = –0.475; *p* = 0.046), and the right caudate zALFF value correlated with the streamlines at the FA value of FCC at the C2 level (r = 0.519; *p* = 0.027) (Figure 3C). A negative correlation was observed between the CSA of the CST at C2 level and the FC between caudate nucleus and right precuneus (r = –0.561; *p* = 0.016), and left PCC (r = –0.573; *p* = 0.013), and right PCC (r = –0.575; *p* = 0.013) (Figures 3D, E).

### 3.5. Clinical scale scores and parameters of the brain and spinal cord

**FIGURE 4:**
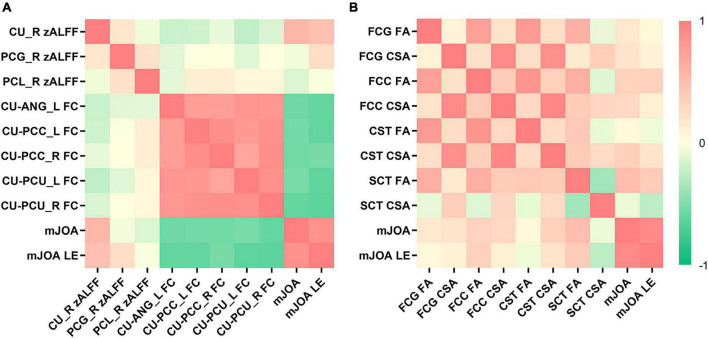
The heatmap demonstrates the relationship between the cerebral functional magnetic resonance imaging (fMRI), spinal diffusion tensor imaging (DTI) parameters and clinical scale scores. **(A)** Patients’ modified Japanese Orthopedic Association (mJOA) and mJOA lower extremity scores were positively correlated with caudate z-score transformation amplitude of low-frequency fluctuations (zALFF). Patients’ mJOA and mJOA lower extremity scores were negatively correlated with the FC between the caudate nucleus and left angular gyrus, bilateral precuneus, and bilateral PCC. **(B)** Patients’ mJOA were positively correlated with FA of the SCT at C2 level. CU, caudate nucleus; ANG, angular gyrus; PCU, precuneus; PCC, posterior cingulate cortex; FCG, fasciculus gracilis; FCC, fasciculus cuneatus, CST, lateral corticospinal tract; SCT, spinocerebellar tract; LE, lower extremity.

A positive correlation was observed between patients’ mJOA and caudate zALFF values (r = 0.560; *p* < 0.001) and FA values of SCT (r = 0.507; *p* = 0.032) at the C2 level, as well as a correlation between mJOA lower extremity scores and caudate zALFF values (Spearman’s ρ = 0.489; *p* = 0.004) (Figures 4A, B). No significant correlation between the symptom duration and parameters of spinal tracts was noted (*P* > 0.05).

Patients’ mJOA was negatively correlated with the FC between the caudate nucleus and the left angular gyrus (r = -0.577; *p* < 0.001), bilateral (left, r = –0.536; *p* = 0.002, right, r = –0.616; *p* < 0.001) precuneus, and bilateral (left, r = –0.561; *p* < 0.001, right, r = –0.562; *p* < 0.001) PCC (Figure 4A). A similar correlation was observed between the mJOA lower extremity scores and the FC between the caudate nucleus and the left angular gyrus (Spearman’s ρ = –0.619; *p* < 0.001), bilateral (left, Spearman’s ρ = –0.637; *p* < 0.001, right, Spearman’s ρ = –0.649; *p* < 0.001) precuneus, and bilateral (left, Spearman’s ρ = –0.619; *p* < 0.001, right, Spearman’s ρ = –0.546; *p* = 0.001) PCC (Figure 4A).

## 4. Discussion

The current study explored the changes in the brain and spinal cord in CSM patients with gait instability. We found that the zALFF of the right caudate nucleus, right postcentral gyrus, and right paracentral lobule were significantly different among HC, nPT, and aPT groups, while zALFF value of the caudate nucleus was the only significantly different region between nPT and aPT groups. The FC between the caudate nucleus and the left angular gyrus, bilateral precuneus and bilateral PCC were also increased in aPT. There was no difference in spinal tract parameters at the C2 level between HC, nPT and aPT. The FA value of FCG was negatively correlated with the paracentral lobule’s zALFF, and FA value of FCC was positively correlated with the caudate zALFF. The CSA of the CST was negatively correlated with the FC between right caudate nucleus and right precuneus and bilateral PCC.

### 4.1. The caudate nucleus

The caudate nucleus, as part of the basal ganglia, is involved in somatic locomotion. The gait and balance had a significant correlation with the volume of the caudate nucleus in patients with white matter hyperintensities (Macfarlane et al., 2015). Similar results were also found in the studies of Parkinson’s disease and cognitive impairment (Rosso et al., 2017; Surkont et al., 2021). When healthy volunteers move through a narrow space, their caudate nucleus is also activated (Marchal et al., 2019). The abnormal activity of muscles was associated with gait instability in CSM (Haddas et al., 2019). Locomotion is a complex behavior that involves the coordinated activation of a large number of muscles. The initiation of locomotion is connected with many brain regions, including the cerebral cortex, the basal ganglia, the midbrain and the hindbrain (Kiehn, 2016). Previous studies also reported that the volume and function of the basal ganglia were changed in CSM (Woodworth et al., 2019; Zhao et al., 2020). In the present study, we observed that the right caudate zALFF value in aPT was higher than the value in HC, and lower than that in nPT, and it was the only significantly different region between nPT and aPT. Moreover, caudate activity was positively correlated with patients’ mJOA lower extremity scores. Hence, we consider that the caudate nucleus might be related to the generation of gait instability in CSM.

### 4.2. FC between the caudate nucleus and default mode network

The default mode network (DMN) is a large-scale brain network that involves memory processes, conceptual processing and emotion processing (Smallwood et al., 2021). A previous study revealed that the activity of DMN was changed, as well as FC between DMN and other brain regions in CSM (Zhao et al., 2020). In the present study, the aPT exhibited enhanced FC between the caudate nucleus and the left angular gyrus, bilateral precuneus, and bilateral PCC, which constitute a vital section of the DMN. The angular gyrus is closely related to action-feedback monitoring and locomotion (Baarbe et al., 2021), and the precuneus was also associated with walking speed and ability of across obstacles (Gonzales et al., 2019). Duan et al. (2012) reported that increased FC between the caudate nucleus and DMN (including the PCC and angular gyrus) could enhance the concentration. The CSM patients with gait instability might need to pay more attention to their locomotion. Therefore, the aPT group exhibited higher FC between the caudate nucleus and DMN. In addition, the significantly different FC were negatively correlated with the mJOA lower extremity scores in current study, we speculated the strengthening FC between caudate nucleus and DMN was aimed at maintaining gait stability in CSM.

### 4.3. Parameters of spinal tracts

In the current study, no significantly different FA value or CSA of tracts at the C2 level were observed between the HC, nPT, and aPT group, which was not consistent with previous studies (Kerkovsky et al., 2012; Chen et al., 2022). In CSM, degeneration of the spinal cord above the compressed level is correlated with Wallerian degeneration (Tu et al., 2021). Its effect was limited by the distance and lesion severity, and degeneration was less pronounced with increasing distance from the lesion (Azzarito et al., 2020). The majority of patients in our study had mild or moderate myelopathy, which may account for the difference. Another reason is that previous studies measured the spinal cord parameters manually, while the present study was performed with the Spinal Cord Toolbox. Therefore, our method can decrease manual measurement errors.

The main difference between nPT and aPT was gait instability in our study. Previous studies revealed that FCG, FCC, CST and SCT were associated with locomotion and gait (Yasuda et al., 1993; Fink, 2013; Chalif et al., 2022; Smith et al., 2022), while there was no difference in these spinal tract parameters at C2 the level between nPT and aPT in the present study. Thus, we thought these spinal tracts microstructures at C2 level might not be correlated to the gait instability in CSM.

The present study found no significant correlation between the symptom duration and parameters of spinal tracts. However, the existing literature on the relationship between symptom duration and spinal cord parameters seemed inconsistent. Some studies found no correlation between symptom duration and DTI parameters (Kara et al., 2011; Gohmann et al., 2019), whereas Wu et al. (2020) reported a statistical correlation. The spinal cord compression often preceded the onset of symptoms and signs (Bednarik et al., 2004). Furthermore, the parameters of spinal cord in asymptomatic patients had changed (Kerkovsky et al., 2012). It was not easy to identify the specific time of spinal cord compression since the CSM duration was based on the earliest incidence of clinical symptoms, which might account for our results.

### 4.4. Correlation between the brain and spinal cord

In recent years, there have been many studies on the functional changes in the brain and spinal cord in CSM. Kuang and Zha (2019). reported that zALFF values were increased in the superior frontal gyrus in CSM patients, and the FC values between changed region and somatosensory cortex were increased. Zhao et al. (2022b) proved that zALFF values of the precentral gyrus could predict the prognosis of CSM. Moreover, the FA value of CST and posterior columns (including FGC and FCC) were decreased, which was related to clinical scale scores (Grabher et al., 2016). These studies suggested that the brain and spinal cord might change together.

The spinal cord is a continuation of the brain, and its function is strongly correlated (Vahdat et al., 2020). A recent animal study reported that reduced inflammation in the spinal cord was related to improved neuronal survival in the brain and neurological recovery (Li et al., 2020). In a CSM study, Zhao et al. (2022a) found that FA of posterior cervical spinal cord and FC of the somatosensory cortex were relevant. The present study showed that the FA value of FCG was negatively correlated with the paracentral lobule’s zALFF, and FA value of FCC was positively correlated with the caudate zALFF, while CSA of the CST was negatively correlated with the FC between right caudate nucleus and DMN in CSM patients. The FCG and FCC are the ascending tract that leads to critical proprioceptive feedback. The CST is a major descending tract that leads from brain to spinal cord, and it was associated with motor functions. The corticospinal reserve capacity plays a vital role in the remodeling of motor region in CSM (Zdunczyk et al., 2018). The symptoms of CSM include gait instability, hand clumsiness and limb sensory losses. We thought that the correlations between spinal cord and brain function might be related to the clinical symptoms in CSM.

### 4.5. CSM and brain remodeling

As reported, CSM patients showed decreased FC between thalamus and paracentral lobe/precentral gyrus after decompression surgery. Moreover, the decreased FC was positively correlated with upper limb movement in post-operative CSM patients (Peng et al., 2020). In CSM patients, the recovery of upper limb pain was related to a decreased FC between postcentral gyrus and dorsolateral prefrontal cortex (Sawada et al., 2020). All these studies indicated that the brain function in CSM patients had changed, which were also related to the severity and prognosis of CSM. The neurological function was usually improved by recruiting other brain regions in CSM patients (Bhagavatula et al., 2016; Ryan et al., 2018). Judging from the results of our study, the maintenance of gait might be responsible for the recruitment of caudate nucleus, and the zALFF of caudate nucleus might be a new imaging biomarker in CSM since the caudate nucleus was the only significantly different region among HC, nPT and aPT groups. We considered that the FC between the caudate nucleus and DMN may be decreased or normal after the improvement of gait, but it should be explored in our further studies.

**FIGURE 5:**
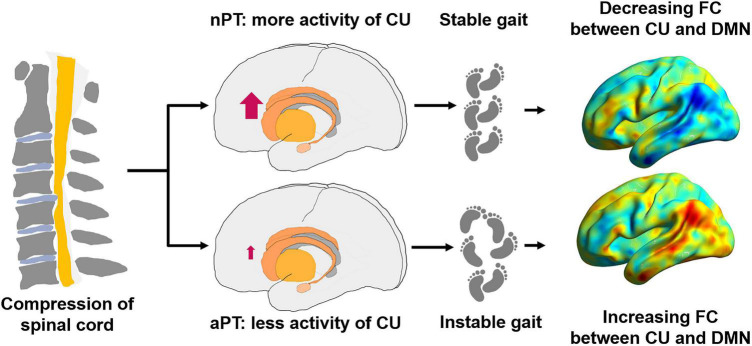
Possible pattern diagram of relationship between the caudate nucleus, the DMN and gait impairment. nPT, normal gait patients; aPT, abnormal gait patients; CU, caudate nucleus; FC, functional connectivity; DMN, default mode network.

Overall, basing on our results, we hypothesized that the brain remodeling might occur before the degeneration of spinal tracts at the C2 level, different activities of the caudate nucleus may be related to the generation of gait instability, while strengthening FC between caudate nucleus and DMN was aimed at maintaining gait stability in CSM (Figure 5), but this conjecture must be explored in follow-up work.

Several limitations in the present study must be acknowledged. Our study was a cross-sectional study that recruited a limited number of participants, and a longitudinal study will be better for observing the evolution of CSM. Another limitation was that the majority of patients had mild or moderate myelopathy. Further studies in a large sample of subjects with more severe disease may be of interest in the future.

## 5. Conclusion

In CSM patients with gait instability, activation of the caudate nucleus likely plays an important role in the generation of gait instability. Furthermore, the strengthening FC between the caudate nucleus and DMN seems to compensate for gait instability. Correlations between spinal cord and brain function might be related to the clinical symptoms in CSM. Our findings contribute to a deeper understanding of gait instability and could have implications on the diagnosis of CSM.

## Data availability statement

The raw data supporting the conclusions of this article will be made available by the authors, without undue reservation.

## Ethics statement

The studies involving human participants were reviewed and approved by Ethics Committee of The First Affiliated Hospital of Anhui Medical University. The patients/participants provided their written informed consent to participate in this study.

## Author contributions

XW and FD had full access to all of the data in the study and take responsibility for the integrity of the data and the accuracy of the data analysis. FD, XW, and YWa conceived and designed the study. XW, YWa, and PS drafting of the manuscript. XW, JC, KZ, YL, SZ, JZ, and SC statistical analysis and graphic design. WJ, TY, PX, and KY collecting the data. YY, YQ, and YWu technical support. FD, PS, and CS study supervision. All authors listed provided the design, made direct contributed to the work and approved the submitted version.

## Footnotes

1
http://www.fil.ion.ucl.ac.uk/spm/

2
https://www.graphpad.com/
